# Nootkatone, a Dietary Fragrant Bioactive Compound, Attenuates Dyslipidemia and Intramyocardial Lipid Accumulation and Favorably Alters Lipid Metabolism in a Rat Model of Myocardial Injury: An In Vivo and In Vitro Study

**DOI:** 10.3390/molecules25235656

**Published:** 2020-11-30

**Authors:** M.F. Nagoor Meeran, Sheikh Azimullah, M Marzouq Al Ahbabi, Niraj Kumar Jha, Vinoth-Kumar Lakshmanan, Sameer N. Goyal, Shreesh Ojha

**Affiliations:** 1 Department of Pharmacology and Therapeutics, College of Medicine and Health Sciences, United Arab Emirates University, Al Ain 17666, UAE; nagoormeeran1985@uaeu.ac.ae (M.F.N.M.); sheikh.azim@uaeu.ac.ae (S.A.); 201509889@uaeu.ac.ae (M.M.A.A.); 2 Department of Biotechnology, School of Engineering & Technology (SET), Sharda University, Plot No.32-34, Knowledge Park III, Greater Noida 201310, Uttar Pradesh, India; niraj.jha@sharda.ac.in; 3 Centre for Preclinical and Translational Medical Research (CPTMR), Central Research Facility (CRF), Faculty of Clinical Research, Sri Ramachandra Institute of Higher Education and Research, Porur, Chennai 600116, Tamil Nadu, India; vinoth.lakshmanan@sriramachandra.edu.in; 4 Department of Biomedical Sciences, College of Medicine, Gulf Medical University, Ajman 4184, UAE; 5 Shri Vile Parle Kelvani Mandal’s Institute of Pharmacy, Dhule 424001, Maharashtra, India; goyal.aiims@gmail.com

**Keywords:** nootkatone, sesquiterpene, bioactive agents, fragrant molecules, essential oils, natural products

## Abstract

In the present study, we assessed whether nootkatone (NKT), a sesquiterpene in edible plants, can provide protection against dyslipidemia, intramyocardial lipid accumulation, and altered lipid metabolism in a rat model of myocardial infarction (MI) induced by subcutaneous injections of isoproterenol (ISO, 85 mg/kg) on days 9 and 10. The rats were pre- and co-treated with NKT (10 mg/kg, p.o.) administered daily for 11 days. A significant reduction in the activities of myocardial creatine kinase and lactate dehydrogenase, as well as non-enzymatic antioxidants, and alterations in lipids and lipoproteins, along with a rise in plasma lipid peroxidation and intramyocardial lipid accumulation, were observed in ISO-treated rats. ISO administration induced alterations in the activities of enzymes/expressions that played a significant role in altering lipid metabolism. However, NKT treatment favorably modulated all biochemical and molecular parameters altered by ISO and showed protective effects against oxidative stress, dyslipidemia, and altered lipid metabolism, attributed to its free-radical-scavenging and antihyperlipidemic activities in rats with ISO-induced MI. Additionally, NKT decreased the accumulation of lipids in the myocardium as evidenced from Oil red O staining. Furthermore, the in vitro observations demonstrate the potent antioxidant property of NKT. The present study findings are suggestive of the protective effects of NKT on dyslipidemia and the underlying mechanisms. Based on our findings, it can be suggested that NKT or plants rich in NKT can be promising for use as a phytopharmaceutical or nutraceutical in protecting the heart and correcting lipid abnormalities and dyslipidemia, which are risk factors for ischemic heart diseases.

## 1. Introduction

Cardiovascular diseases (CVDs) represent one of the primary causes of disability and mortality in industrialized and developing nations due to lifestyle changes and related metabolic disorders; they account for millions of deaths each year with exponential growth expected until 2030 [1]. Among the many manifestations of CVDs, ischemic heart disease (IHD), also known as acute coronary syndrome, refers to a wide array of clinical conditions including unstable angina, myocardial injury, and myocardial infarction (MI), caused by a sudden onset of cardiac tissue ischemia secondary to impaired blood flow. MI is one of the major events which occurs due to scarcity of coronary blood supply, which damages heart muscles with characteristic changes in the cardiac function, along with histological and morphological changes [2]. Epidemiological data revealed that hyperlipidemia-mediated atherosclerosis, characterized by high levels of lipids in the bloodstream, is directly proportional to the risk of IHD. There are various risk factors for CVDs, especially acute MI, which include excessive fat intake, physical inactivity, and stress [3]. Accumulating evidence indicates that hyperlipidemia, altered lipid metabolism, has a fundamental role in the pathology of MI. Lipid accretion in the myocardium has been associated with MI induced by isoproterenol (ISO), a synthetic catecholamine and a β-adrenergic agonist, at higher doses to the laboratory animals, which mimics human MI [4]. The supramaximal physiological doses of ISO trigger oxidative stress and play a central role in the altered myocardial contraction, as well as energy and lipid metabolism [5,6] and they elevate the levels of circulatory lipids [7]. Furthermore, ISO elicits lipolysis in the myocardium and enhances the amount of circulatory low-density lipoprotein cholesterol (LDL-C) responsible for accumulation in the coronary arteries, supporting the development of coronary artery disease [8,9]. The generation of free radicals by ISO promotes atherosclerotic lesions via the formation of oxidized low-density lipoprotein cholesterol (oxidized LDL cholesterol) from LDL cholesterol, which is one of the therapeutically important underlying causes of MI [10]. ISO-induced biochemical changes, especially impaired lipid metabolism, are analogous to the changes which occur in humans [11]. Thus, correcting the altered lipid metabolism attracted enormous attention for therapeutic targeting and generated special interest in exploring natural dietary agents that may improve lipid metabolism, as well as attenuate dyslipidemia and cardiac hypertrophy. Additionally, these further alter the functional properties of the cardiac cell membrane and lead to decreased myocardial contractility, arrhythmias, and cell death following occlusion of the coronary arteries and sequelae [12].

The ISO-induced experimental model of MI is technically reliable, simple, and reproducible with low mortality compared to other models such as coronary artery ligation, which involves various surgical procedures including thoracotomy, coronary ligation, ventilator support, and postoperative analgesia [13]. This is one of the most popular models to evaluate agents and drugs, specifically, natural products, including plant extracts and plant-derived bioactive compounds, known as phytochemicals, for their therapeutic potential and pharmacological mechanisms in IHDs. Many of the dietary phytochemicals have been shown to be associated with a decreased risk of the development of diabetes, neurodegenerative diseases, cancer, and CVDs [14]. Despite many synthetic drugs being currently available for IHD, the need of natural dietary therapeutic agents is gaining attention due to rising confidence in complementary and alternative medicine along with lesser side effects. Many of the dietary phytochemicals were shown to be related to lessened menace for the development of neurodegenerative, cardiovascular, and cardiometabolic diseases [14].

**Figure 1:**
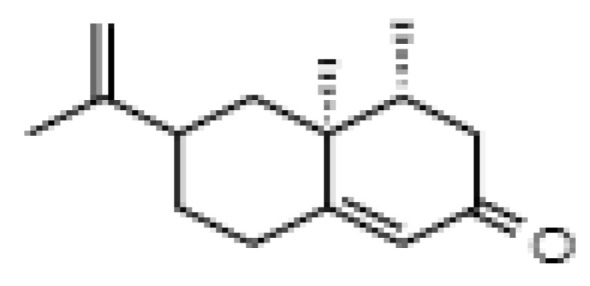
Structure of nootkatone (NKT).

Among numerous phytochemicals of dietary plant origin, nootkatone (NKT) (Figure 1), a natural sesquiterpene present in grapefruit, received attention for its plausible health benefits and pharmacological properties such as free-radical-scavenging, anti-inflammatory, and antiapoptotic activities [15,16]. Furthermore, NKT was found to prevent diet-induced obesity by activating liver and muscle AMP-activated protein kinase (AMPK), promoting energy metabolism [17]. Circulating lipids and lipoproteins are involved in the pathology of MI. To counter the risk of MI, treatment strategies should focus on maintaining circulatory lipids and lipoproteins. The ability of NKT in countering high-fat diet-induced obesity roused our interest to investigate the therapeutic potential of NKT in a rat model of myocardial injury induced by ISO. According to its properties, the present study aimed to investigate whether NKT can provide protection against the altered lipid profile induced by ISO in rats. As oxidative stress, dyslipidemia, and altered lipid metabolism are the most common risk factors of MI, and there is not sufficient scientific evidence available on the possible beneficial properties of NKT against these parameters, we evaluated NKT in rats with ISO-induced MI. Furthermore, to evaluate the antioxidant capacity of NKT, we also assessed its action on reducing power in an in vitro study. The present study appears to be the first demonstrating the protective effects of NKT against dyslipidemia and altered lipid metabolism in ISO-induced MI.

## 2. Results

### 2.1. Effect of NKT on Cardiac Enzyme Markers

**Figure 2:**
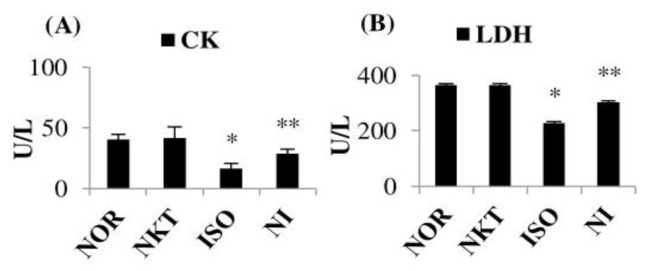
Effect of NKT on cardiac markers: concentrations of (**A**) creatine kinase (CK) and (**B**) lactate dehydrogenase (LDH) in the myocardium. Each column is the mean ± standard error of the mean (SEM) for eight rats in each group; * *p* < 0.05 as compared to the normal control (Group-I), ** *p* < 0.05 as compared to the isoproterenol (ISO) control (Group-III) (Duncan’s multiple range test; DMRT).

ISO-induced MI in rats resulted in a significant (*p* < 0.05) decrease in the concentrations of CK and LDH in the heart in comparison with normal control rats. However, pre- and cotreatment with NKT prevented the injury-induced release of CK and LDH into the serum, as evidenced by the significant (*p* < 0.05) increase in the concentrations of CK and LDH in the heart of ISO-treated rats compared to ISO control rats (Figure 2A,B).

### 2.2. Effect of NKT on Plasma Lipid Peroxidation and Non-Enzymatic Antioxidants

**Figure 3:**
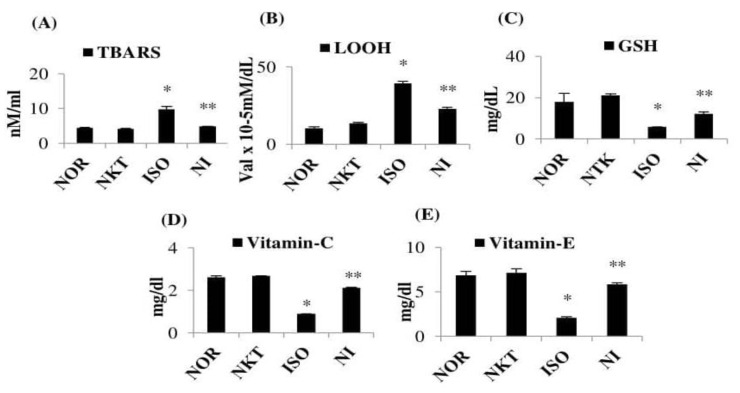
Effect of NKT on plasma lipid peroxidation and non-enzymatic antioxidants: (**A**–**E**) levels of plasma lipid peroxidation products and non-enzymatic antioxidants. Each column is the mean ± SEM for eight rats in each group; * *p* < 0.05 as compared to the normal control (Group-I), ** *p* < 0.05 as compared to the ISO control (Group-III) (DMRT).

ISO injections in rats caused a significant (*p* < 0.05) increase in the levels of plasma lipid peroxidation products such as TBARS and LOOH with a significant (*p* < 0.05) decrease in the levels of plasma non-enzymatic antioxidants such as GSH, vitamin C, and vitamin E compared to normal control rats. Pre- and cotreatment with NKT showed a significant (*p* < 0.05) decrease in the levels of plasma lipid peroxidation products with a significant (*p* < 0.05) increase in the plasma non-enzymatic antioxidants in comparison with ISO control rats (Figure 3A–E).

### 2.3. Effect of NKT on Heart Weight and Left-Ventricular Weight

**Figure 4:**
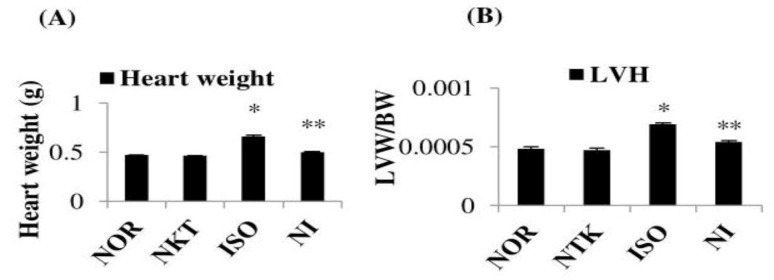
Effect of NKT on heart weight and left-ventricular hypertrophy (LVH): (**A**) heart weight and (**B**) LVH in the myocardium. Each column is the mean ± SEM for eight rats in each group; * *p* < 0.05 as compared to the normal control (Group-I), ** *p* < 0.05 as compared to the ISO control (Group-III) (DMRT).

ISO-induced MI in rats resulted in a significant (*p* < 0.05) increase in heart weight (cardiac hypertrophy) and left-ventricular weight/body weight (left-ventricular hypertrophy) in comparison with normal control rats. Pre- and cotreatment with NKT significantly (*p* < 0.05) decreased the heart weight (cardiac hypertrophy) and left-ventricular weight/body weight (LVH) in ISO-treated rats compared to ISO control rats (Figure 4A,B).

### 2.4. Effect of NKT on the Levels/Concentrations of Lipids in the Serum and Heart

**Figure 5:**
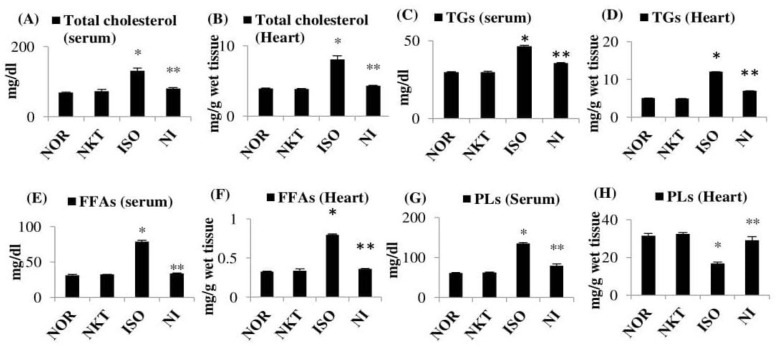
Effect of NKT on the levels of lipids/concentrations of lipids: (**A**–**H**) levels/concentrations of total cholesterol, triglycerides (TGs), free fatty acids (FFAs), and phospholipids (PLs) in the serum and heart. Each column is the mean ± SEM for eight rats in each group; * *p* < 0.05 as compared to the normal control (Group-I), ** *p* < 0.05 as compared to the ISO control (Group-III) (DMRT).

ISO-induced MI in rats resulted in a significant (*p* < 0.05) increase in the levels/concentrations of serum and heart total cholesterol, TGs, and FFAs compared to normal control rats. Furthermore, the levels of serum PLs were significantly (*p* < 0.05) increased and the concentration of PLs in the heart was significantly (*p* < 0.05) decreased in ISO-induced rats compared to normal control rats. Treatment with NKT showed a significant (*p* < 0.05) decrease in the levels/concentrations of serum and heart total cholesterol, TGs, and FFAs with a significant (*p* < 0.05) rise in PL levels compared to ISO-treated rats (Figure 5A–H).

### 2.5. Effect of NKT on Alteration in the Levels of Lipoproteins

**Figure 6:**
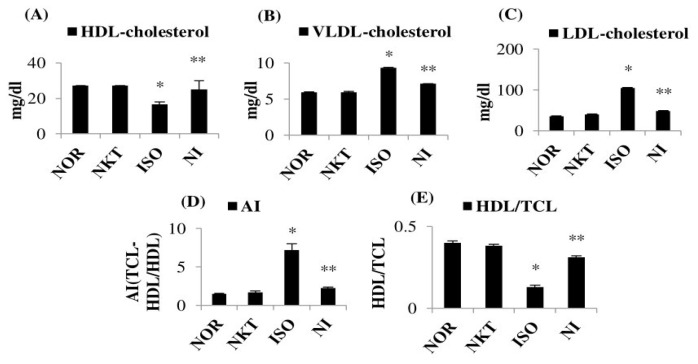
Effect of NKT on lipoproteins, AI, and high-density lipoprotein (HDL)/TCL ratio: (**A**–**C**) levels of serum lipoproteins (HDL cholesterol, low-density lipoprotein (LDL) cholesterol, and very-low-density lipoprotein (VLDL) cholesterol); (**D**,**E**) AI and HDL/TCL ratio. Each column is the mean ± SEM for six rats in each group; * *p* < 0.05 as compared to the normal control (Group-I), ** *p* < 0.05 as compared to the ISO control (Group-III) (DMRT).

ISO caused a significant (*p* < 0.05) alteration of lipoproteins (enhanced serum LDL cholesterol and VLDL cholesterol, and decreased HDL-cholesterol) compared to normal control rats. Furthermore, ISO significantly (*p* < 0.05) decreased the HDL/total cholesterol (TCL) ratio and augmented atherogenic index (AI) in comparison with normal rats. However, treatment with NKT significantly (*p* < 0.05) normalized the lipoprotein levels in comparison with ISO control rats (Figure 6A–E).

### 2.6. Effect of NKT on Free Cholesterol and Cholesterol Esters

**Figure 7:**
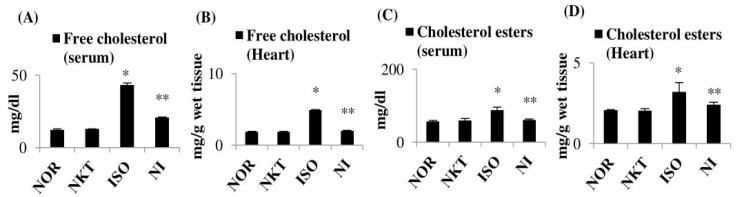
Effect of NKT on free cholesterol and cholesterol esters in the serum and heart: (**A**–**D**) the levels/concentrations of free cholesterol and cholesterol esters in the serum and heart. Each column is the mean ± SEM for eight rats in each group; * *p* < 0.05 as compared to the normal control (Group-I), ** *p* < 0.05 as compared to the ISO control (Group-III) (DMRT).

ISO caused a significant (*p* < 0.05) increase in serum and heart free cholesterol and cholesterol esters compared to normal control rats. However, treatment with NKT significantly (*p* < 0.05) normalized the free cholesterols and cholesterol esters in the serum and heart with significantly (*p* < 0.05) lessened AI in comparison with ISO control rats (Figure 7A–D).

### 2.7. Effect of NKT on Lipid Marker Enzymes in the Plasma and Liver of Rats

**Figure 8:**
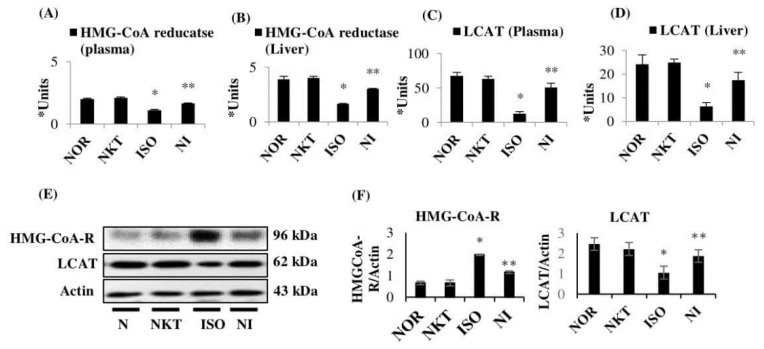
Effect of NKT of lipid marker enzymes in the plasma and liver: (**A**,**B**) activity of 3-hydroxy-3-methylglutaryl coenzyme-A reductase (HMG-CoA) reductase in the plasma and liver. Each column is the mean ± SEM for eight rats in each group; * *p* < 0.05 as compared to the normal control (Group-I), ** *p* < 0.05 as compared to the ISO control (Group-III) (DMRT). In group III, the lower ratio of HMG-CoA/mevalonate indicates higher enzyme activity and, in Group IV, a higher ratio of HMG-CoA/mevalonate indicates lower enzyme activity; (**C**,**D**) activity of lecithin cholesterol acyltransferase (LCAT) in the plasma and liver. Each column is the mean ± SEM for eight rats in each group; * *p* < 0.05 as compared to the normal control (Group-I), ** *p* < 0.05 as compared to the ISO control (Group-III) (DMRT). * Units: μM of cholesterol esterified/h/mL for plasma LCAT and μM of cholesterol esterified/h/mg protein for liver; (**E**,**F**) Western blotting analysis of HMG-CoA-R and LCAT in the liver and the densitometric analysis. Columns not sharing a common symbol (*, **) differ significantly from each other (* *p* < 0.05 vs. normal control, ** *p* < 0.05 vs. ISO control).

A significant (*p* < 0.05) rise in the activity of HMG-CoA-R (i.e., lowered HMG-CoA/mevalonate ratio), along with a significant (*p* < 0.05) reduction in the activity of LCAT, was seen in the plasma and liver of ISO-treated rats in comparison with normal rats. Treatment with NKT produced a significant (*p* < 0.05) reduction in HMG-CoA-R activity (i.e., increased HMG-CoA/mevalonate ratio) in addition to a significant (*p* < 0.05) rise in activity of LCAT in the plasma and liver of ISO-treated rats in comparison with ISO control rats (Figure 8A–D). A significant (*p* < 0.05) increase in the protein expression of HMG-CoA-R, along with a significant (*p* < 0.05) decrease in the protein expressions of LCAT, was seen in the liver of ISO-treated rats in comparison with normal rats. Treatment with NKT produced a significant (*p* < 0.05) reduction in the protein expression of HMG-CoA-R, in addition to a significant (*p* < 0.05) increase in the expression of LCAT, in the liver of ISO-treated rats in comparison with ISO control rats (Figure 8E,F).

### 2.8. Effect of NKT on Intramyocardial Lipid Accumulation in the Myocardium

**Figure 9:**
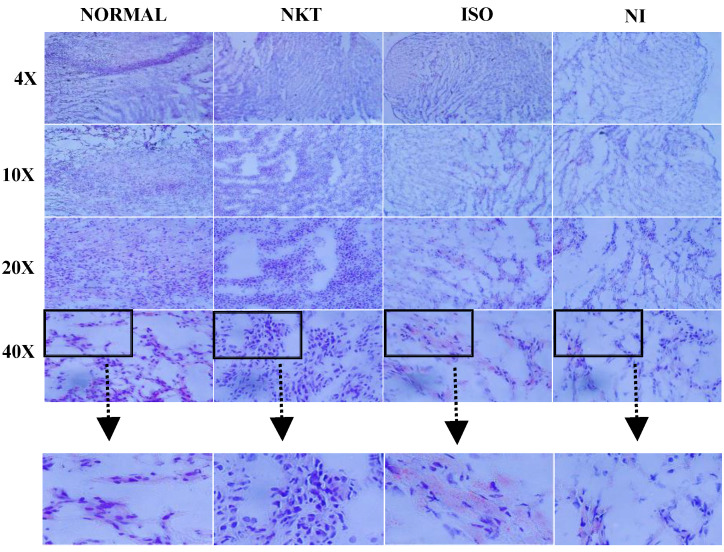
Effect of NKT on lipid accumulation in the myocardium (Oil red O staining). Normal control rat’s heart revealing normal myocardial architecture without lipid accumulation; NKT alone-treated rat’s heart also showing normal intact architecture of myocardium without lipid accumulation; ISO-induced rat’s heart showing extensive lipid accumulation compared to normal control rats; NKT-treated ISO-induced MI in rat’s heart showing reduced lipid accumulation compared to ISO-induced rats.

The hearts of normal and NKT alone-treated rats showed no intramyocardial lipid accumulation. However, rats treated with ISO revealed severe intramyocardial lipid accumulation. Administration of NKT to ISO-induced MI in rats protects the myocardium as evidenced by less lipid accumulation (Figure 9).

### 2.9. The In Vitro Reducing Power of NKT

**Figure 10:**
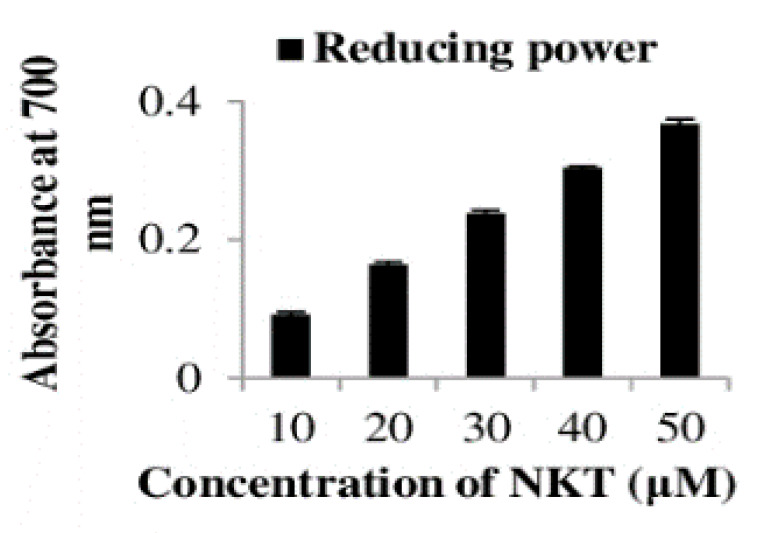
The in vitro reducing power of NKT. The columns are the average of experiments in triplicate.

The reducing power of NKT at varying concentrations was measured in an in vitro setting (Figure 10). NKT shows potential reducing power with higher concentrations. The reducing power of NKT measured at 700 nm for the different studied concentrations (10, 20, 30, 40, and 50 µM) was found to be 0.091, 0.164, 0.237, 0.303, and 0.367, respectively. The results demonstrate that NKT is a potent reductant, and the reducing actions of NKT correlate with its antioxidant property.

## 3. Discussion

Myocardial enzymes are biomarkers of heart function, and release of these enzymes into circulation after ISO administration demonstrates altered integrity of the plasma membrane due to sarcolemmal damage, which renders the membrane leaky [18]. Decreased activities of CK and LDH in the myocardium clearly reflect the increased CK and LDH activities in the serum. Excessive free radicals produced by ISO damage the lipophilic cell membrane, as indicated by the elevated lipid peroxidation in terms of TBARS and LOOH. Increased plasma lipid peroxidation is a crucial event in the institution and development of MI and its complications. Diminished concentrations of plasma GSH, an intracellular free-radical scavenger, is believed to be due to its counteractivity against free radicals in oxidative stress triggered by ISO [19]. Moreover, diminished levels of vitamin C and E are attributed to their increased utilization against reactive oxygen species (ROS) [20]. The increase in heart weight occurs due to a rise in edematous intramuscular space following cardiomyocyte injury and swelling, along with the invasion of the injured tissues by defensive inflammatory cells. A significant growth in left-ventricular mass during chronic workload represents LVH, which is considered a critical factor in impairing contractile function and subsequent heart failure [21]. NKT prevents the leakage of cardiac marker enzymes and salvages myocardial tissues while inhibiting the excessive workload of the myocardium due to its potent free membrane-stabilizing properties attributed to its antioxidant and radical scavenger action.

The risk factors of CVDs have long been studied in order to gain insight into the epidemiology, pathophysiology, and therapy of MI. The lipid profile is commonly considered as an indication of the tissue metabolism and the porosity of the cell layer to different particles, and it depends upon the synthesis of lipids [22]. High levels of lipids and alterations in the levels of lipoproteins in the serum are associated with an increased risk of CVDs [11]. Maintaining the homeostasis of cellular cholesterol is a crucial mechanism for the prevention of MI. The increased myocardial cholesterol content in ISO-induced rats is due to increased uptake of LDL-cholesterol from the blood by myocardial membranes. Maintaining cholesterol equilibrium is a key factor in the preclusion of CVDs. Cholesterol aggregation triggers atherogenesis and hypercholesterolemia that leads to the advancement of CVDs. Lipid accumulation in circulation is a major risk factor in MI. This could be due to rapid FFA mobilization from fat deposits, which are associated with myocardial damage. Abnormal concentrations of lipids produce free radicals, followed by lipid peroxidation, which is responsible for irreversible myocardial membrane damage and, thus, favors myocardial lipid deposition [21]. Increased serum cholesterol levels are associated with myocardial damage and augmented uptake of LDL cholesterol in the myocardium from the blood. Additionally, amplified lipid biosynthesis is one of the major reasons for increased myocardial cholesterol content in ISO-induced MI [9]. Increased myocardial cholesterol alters membrane fluidity, ionic permeability, and membrane-bound enzymes and promotes PL degradation [23].

The increased TG concentrations during MI are because of an augmented flux of fatty acids and inefficient elimination of plasma VLDL from the circulation [24]. Increased levels of circulatory FFAs have been observed in MI, dyslipidemia, and hypertrophy [25]. This might be due to the decline in FFA oxidation, which can be ascribed to suppressed cardiac function by triggering an irregular heart rhythm and loss of synergy in heart function, which may lead to myocardial injury [21]. Phospholipids are widely present in cell membranes, and they are important for enhanced fortification of cellular reliability, microviscosity, and endurance. Increased PL deprivation with a higher ratio of cholesterol/PLs might result in cell injury, membrane dysfunction, and cell death. Phospholipid degradation by phospholipase A2 during lipid peroxidation is the crucial mechanism behind the insufficient phospholipid levels in the myocardium. ISO increased the activity of phospholipase A2 in the myocardium [26]. Increased PL degradation leads to cell death, as evidenced by the TBARS accumulation and loss of extractable PLs and their polyunsaturated acyl groups [23]. A rise in AI indicates the potential risk of endothelial dysfunction and CVDs [27]. High concentrations of blood cholesterol, particularly LDL and VLDL cholesterol, with low HDL cholesterol concentrations are important risk factors for developing CVDs [28]. A negative correlation exists between total body cholesterol and HDL cholesterol levels. The concentration of LDL cholesterol correlates positively, whereas HDL cholesterol correlates inversely to the development of coronary heart disease. Monitoring LDL cholesterol is preferred over total cholesterol, because it is more closely related to risk [29]. HDL cholesterol’s protective function has been attributed to its active participation in the reverse transport of cholesterol by inhibiting the uptake of LDL by the arterial wall, and it facilitates cholesterol transport to the liver where it is metabolized and discharged from the body [30]. NKT treatment prevents the risk of MI by preventing dyslipidemia and by decreasing AI and increasing HDL-C/total cholesterol ratio in ISO-induced MI.

A significant rise in the activity of HMG-CoA-R, a rate-limiting enzyme for cholesterol biosynthesis, leads to increased generation of cholesterol, which results in the formation of foam cells, a prerequisite in the initiation and progression of atherosclerosis [31]. There are several medications available for treating the hypercholesterolemic complications, including HMG-CoA-R inhibitors such as statins, which maintain cholesterol metabolism in the body via downregulating HMG-CoA-R activity [32]. Increased lipid peroxidation is the main factor involved in increased HMG-CoA-R activity in the plasma and liver of rats challenged with ISO injections. LCAT is an HDL-related protein, which possesses a crucial role in extracellular cholesterol digestion and transport. HDL cholesterol is the main substrate for LCAT for cholesterol esterification and intoxication. It is responsible for the formation of most cholesterol esters found in circulation. The decreased activity of LCAT inhibits cholesterol esterification in ISO-induced myocardial infarcted rats. This leads to increased levels of lipids and lipoproteins in circulation, which lead to high risk of MI [21]. LCAT promotes cholesterol esterification on the surface of HDL, giving protection against MI. Increased oxidative stress diminishes the activity of LCAT, which leads to a slowdown of cholesterol esterification in ISO-induced MI in rats [33]. Altered lipoprotein levels might be due to the LCAT inhibition by ISO-induced oxidative stress [23]. NKT treatment appears to regulate the synthesis of cholesterol by inhibiting the activity of HMG-CoA-R and by enhancing the HDL cholesterol turnover through increasing the activity of LCAT in ISO-triggered hyperlipidemia in rats, which is suggestive of the antihyperlipidemic property of NKT.

Adrenergic activation exerts positive inotropic and chronotropic effects on the myocardium and regulates lipid metabolism [34,35]. Intracellular lipid accumulation may serve as a substrate for lipid peroxidation, and the subsequent products can destroy intracellular proteins and disrupt the biomembrane integrity, resulting in the disturbance of ion transport [36,37]. NKT prevents altered lipid metabolism and prevents the accumulation of intramyocardial lipids by virtue of its potent antihyperlipidemic property. The reducing ability of a molecule reflects its antioxidant potential [38]. These agents have the potential to attenuate oxidative stress by reducing ROS generation, thereby diminishing the institution, progression, and development of various diseases and complications. The agents which possess reducing power cause a reduction in the transformation of the Fe^3+^/ferricyanide complex to its ferrous form [39]. The potent reducing power of NKT clearly indicates its potent antioxidant activity.

In conclusion, the protective actions of NKT are ascribed to its action on lipids and lipoproteins by virtue of its potent antioxidant and antilipidemic properties. NKT attenuates lipid peroxidation, enhances antioxidant defense mechanisms, and favorably modulates HMG-CoA reductase and LCAT, thereby preventing hyperlipidemia, altered lipoproteins, and hypertrophy in a rat model of MI induced by ISO. The favorable modulation of the lipid profile and biochemical findings are further supported by the potent reducing power of NKT observed in the in vitro study.

## 4. Materials and Methods

### 4.1. Drugs and Chemicals

Isoprenaline hydrochloride (catalog number: I5627), nootkatone (purity: 98%) (catalog number: W316620), thiobarbituric acid (catalog number: T5500), phosphotungstic acid (catalog number: 79690), 1,1′,3,3′-tetra methoxy propane (108383), xylenol orange (33825), 2,4-dinitro phenylhydrazine (42215), and 2,2′-dipyridyl (D216305) were procured from Sigma-Aldrich, St. Louis, MO, USA. In the present study, the other laboratory chemicals and reagents utilized were of required analytical grade.

### 4.2. Experimental Animals

To conduct the animal experiments, laboratory-bred, male Wistar rats (200–250 g) were obtained from the animal research facility of the College of Medicine and Health Sciences, United Arab Emirates University. The rats were housed in polycarbonate cages (47 × 34 × 20 cm^3^) (four rats per cage) lined with husk, renewed every 24 h under a 12 h light/dark cycle at around 22 °C with 50% humidity. The animals were divided into four groups containing 15 animals each. Prior to the start of the experiment, the rats were acclimatized for a week to the laboratory conditions. Each animal had free access to the commercially available rodent chow diet and purified water ad libitum. The rats were administered either ISO or the test drug, NKT, at a fixed time, and they were euthanized during the daytime. Approval for the experimental protocol and procedure adopted in the conduct of the animal experiments was obtained from the Animal Ethics Committee of the United Arab Emirates University, United Arab Emirates.

### 4.3. Induction of Experimental MI in Wistar Rats

To induce MI in rats, ISO (85 mg/kg body weight), prepared freshly before the injection by dissolving in saline, was injected subcutaneously into rats for 2 days subsequently following an interval of 24 h [19]. The induction of MI was confirmed by reduced activity of creatine kinase (CK) and lactate dehydrogenase (LDH), the diagnostic markers of MI. The design consisted of four study groups wherein each study group contained 15 rats. The rats in group I were given normal saline only and designated as the normal control group. The rats in group II were treated orally with NKT (10 mg/kg body weight) daily for a period of 11 days and designated as the NKT control group. The rats in group III were subcutaneously injected with ISO (85 mg/kg body weight) for 2 subsequent days at an interval of 24 h on days 9 and 10 and designated as the ISO-induced MI group. The rats in group IV were orally pre- and cotreated with NKT (10 mg/kg body weight) daily for a period of 11 days and subcutaneously injected with ISO (85 mg/kg body weight) for 2 days at an interval of 24 h on days 9 and 10 and designated as the NKT-treated MI group. Twenty-four hours after the second dose of ISO injection, the rats from each experimental group were anaesthetized using an intraperitoneal injection of pentobarbital sodium (60 mg/kg) and then sacrificed by cervical decapitation; then, the heart and liver were excised immediately, rinsed in ice-chilled saline, and snap-frozen. The blood was collected in tubes with and without anticoagulant to get plasma and serum, respectively. The heart and liver tissues were weighed and homogenized in suitable buffers following their weight for the estimation of different biochemical parameters.

### 4.4. Biochemical Estimations

#### 4.4.1. Estimation of the Cardiomyocyte Injury Marker Enzymes

The levels of CK and LDH in heart samples were estimated using commercially available assay kits and employing the Chemistry Analyzer (Vet Test 8008, Wetherby, UK).

#### 4.4.2. Determination of Plasma Thiobarbituric Acid-Reactive Substances (TBARS)

The amount of plasma TBARS was estimated using the method of Yagi [40]. To 0.5 mL of plasma, 4.0 mL of 0.083 N sulfuric acid and 0.5 mL of phosphotungstic acid (10%) were added. The reaction mixture was mixed gently and incubated for 5 min at room temperature. After incubation, the mixture was centrifuged at 3000× *g* for 10 min. The sediment was thoroughly mixed with 2.0 mL of sulfuric acid (0.083 N) and 0.3 mL of 10% phosphotungstic acid. Then, the mixture was shaken well and centrifuged at 3000× *g* for 15 min. The sediment was again suspended in 4.0 mL of distilled water and 1.0 mL of thiobarbituric acid reagent. The mixture was kept at 95 °C for 1 h. The tubes were cooled followed by the addition of 5.0 mL of *n*-butanol. After vigorous shaking, the tubes were centrifuged for 20 min. The color occurring in the butanol layer was read at 530 nm. Standard malondialdehyde (1–5 nM) in 4.0 mL volume and 4.0 mL of distilled water (blank) were also processed.

#### 4.4.3. Estimation of Plasma Lipid Hydroperoxides (LOOH)

The levels of plasma LOOH were estimated using the standard method of Jiang et al. [41]. First, 1.8 mL of the Fox reagent was added to 0.2 mL of plasma and mixed well. The mixture was incubated for 30 min at room temperature, and the developed color was read at 560 nm.

#### 4.4.4. Estimation of Plasma reduced glutathione (GSH)

The levels of plasma GSH were analyzed using the method of Ellman [42]. First, 0.2 mL of plasma was precipitated with 2.0 mL trichloro acetic acid (5%). The mixture was then centrifuged, and the supernatant was collected. Around 1 mL of the supernatant was mixed with 0.5 mL of Ellman’s reagent and 3.0 mL of phosphate buffer (0.2 M, pH-8.0). The yellow color developed was read at 412 nm.

#### 4.4.5. Estimation of Plasma Vitamin C

The levels of plasma vitamin C were estimated using the method of Omaye et al. [43]. To 0.5 mL of plasma, 1.5 mL of 6% trichloro acetic acid was added. The mixture was shaken well and centrifuged at 3500× *g* for 20 min, and the supernatant was collected. Then, 0.5 mL of the supernatant was mixed with 0.5 mL of dinitro phenylhydrazine reagent. The mixture was incubated at room temperature for 3 h and placed in ice-cold water. Then, 2.5 mL of 85% sulfuric acid was added before incubating for 30 min at room temperature. The color developed was read at 530 nm.

#### 4.4.6. Estimation of Plasma Vitamin E

The levels of plasma vitamin E were estimated using the method of Baker et al. [44]. To 0.5 mL of plasma, 1.5 mL of ethanol and 2.0 mL of petroleum ether were added, before mixing and centrifuging. The supernatant was evaporated to dryness at 80 °C. To this, 0.2 mL of 2,2′-dipyridyl solution and 0.2 mL of ferric chloride were added; then, the solution was mixed well and kept in dark for 5 min before 2.0 mL of butanol was added. The red color developed was read at 520 nm.

#### 4.4.7. Determination of Left-Ventricular Hypertrophy (LVH)

Left-ventricular hypertrophy (LVH) was determined using the standard method of Gupta et al. [45]. The atria and right ventricle were separated along the atrial/ventricular septal wall, and the left-ventricular weight was measured. The ratio between the left-ventricle weight and body weight was used to measure left-ventricular hypertrophy.

#### 4.4.8. Extraction of Lipids in the Heart

The heart tissues were processed to extract lipids using a mixture of chloroform and methanol (2:1, *v*/*v*) following the previously described method of Folch et al. [46]. The tissues were thoroughly rinsed in ice-cold physiological saline and dried by pressing between the folds of filter paper. The samples were homogenized in cold chloroform–methanol (2:1, *v*/*v*), and the contents were extracted after 24 h. The extraction was repeated for four times. The combined filtrate was washed with 0.7% potassium chloride, and the aqueous layer was discarded. The organic layer was made up to a known volume with chloroform and used for various biochemical estimations.

#### 4.4.9. Estimation of Total Cholesterol

The level/concentration of total cholesterol in the serum and heart was estimated using the method of Zlatkis et al. [47]. To 0.5 mL of the dried lipid fraction/0.2 mL of serum, 5.0 mL of ferric chloride–acetic acid reagent was added. After vigorous mixing, 3.0 mL of concentrated sulfuric acid was added and read after 20 min at 560 nm. Standard cholesterol (3–15 µg) was made up to 5.0 mL with the assay reagent to establish a calibration curve along with a blank.

#### 4.4.10. Estimation of Triglycerides (TGs)

The level/concentration of TGs in the serum and heart was estimated using the method of Fossati and Prencipe [48]. To 0.5 mL of the dried lipid fraction/0.2 mL of serum, 0.1 mL of methanol and 4.0 mL of isopropanol were added. Then, 0.4 g of alumina was added before mixing well for 15–20 min. The mixture was centrifuged, and the supernatant was collected. Around 2.0 mL of supernatant was transferred to separate tubes and heated at 65 °C for 20 min. Then, 0.6 mL of the saponification reagent, followed by 0.5 mL of acetyl acetone reagent, was added. After shaking well, the tubes were again heated at 65 °C for 1 h. Triolein standards (8–40 µg) and blanks were treated similarly. After cooling, the absorbance was read at 405 nm.

#### 4.4.11. Estimation of Free Fatty Acids (FFAs)

The level/concentration of FFAs in the serum and heart was estimated using the method of Falholt et al. [49]. To 0.5 mL of the dried lipid fraction/0.2 mL of serum, exactly 1.0 mL of phosphate buffer (pH 6.3), 2.5 mL of copper reagent, and 6.0 mL of extraction solvent were added. The tubes were shaken well for 90 s and kept aside for 15 min. After centrifugation, 3.0 mL of the upper layer was mixed with 0.5 mL of diphenyl carbazide solution. The absorbance was read after 15 min at 550 nm. Phosphate buffer was used as the blank.

#### 4.4.12. Estimation of Phospholipids (PLs)

The level/concentration of PLs in the serum and heart was estimated using the method of Zilversmit and Davis [50]. To 0.5 mL of the dried lipid fraction/0.2 mL of serum, 1 mL of 5 N H_2_SO_4_ was added before incubating until the appearance of a light-brown color. Two to four drops of concentrated HNO_3_ was added, and digestion continued until the disappearance of the brown color. The mixture was cooled, mixed with 1.0 mL of distilled water, and kept in a boiling water bath for 5 min. Lastly, 0.1 mL of amino napthol sulfonic acid and 1.0 mL of 2.5% ammonium molybdate were added. The mixture was made up to 5.0 mL with distilled water, and the absorbance was measured at 660 nm.

#### 4.4.13. Estimation of Lipoproteins

High-density lipoprotein cholesterol (HDL cholesterol), free cholesterol, and cholesterol esters were determined using a standard kit procured from commercial suppliers (Abcam, MA, USA) (catalog number: ab65390). Low-density lipoprotein cholesterol (LDL cholesterol) and very-low-density lipoprotein cholesterol (VLDL cholesterol) were derived following the formula presented below.

VLDL cholesterol = TGs/5(1)

LDL cholesterol = Total cholesterol − (HDL cholesterol + VLDL cholesterol)(2)

#### 4.4.14. Assay of 3-Hydroxy-3-methylglutaryl Coenzyme-A Reductase (HMG-CoA-R)

The ratio of the rate-limiting enzyme in cholesterol metabolism, HMG-CoA-R, to mevalonate was calculated to serve as the enzyme activity index of HMG-CoA-R following the method of Rao and Ramakrishnan [51]. Equal volumes of plasma/liver homogenates and diluted perchloric acid were mixed. The mixture was incubated for 5 min and centrifuged (2000× *g* for 10 min). Then, 1 mL of the filtrate was mixed with 0.5 mL of alkaline hydroxylamine reagent. Finally, 1.5 mL of ferric chloride was added and shaken well for 5 min, and the absorbance was read at 540 nm. A saline–arsenate mixture was treated as the blank. The ratio of HMG-CoA to mevalonate was calculated, where a lower ratio indicates higher enzyme activity and vice versa.

#### 4.4.15. Assay of Lecithin Cholesterol Acyltransferase (LCAT)

The activity of LCAT in the plasma and liver samples was estimated using the method described by Hitz et al. [52]. To 1.0 mL of liver homogenate, 0.5 mL of dextran sulfate (0.2%) was added. The mixture was kept at 4 °C for 15 min and centrifuged at 1750× *g* for 15 min. The supernatant was taken as an enzyme source, and its activity was assayed. To 0.6 mL of the enzyme, 0.6 mL of the substrate was added and shaken well. Then, 0.2 mL of the mixture was mixed with 1 mL of isopropanol, and the remaining mixture was kept at 27 °C for 90 min. The levels/concentrations of serum and heart total cholesterol were estimated using the method of Zlatkis et al. [47]. This represents the amount of free cholesterol present in the test sample at zero time. After 90 min, 0.2 mL of the incubated mixture was mixed with 1.0 mL of isopropanol, and the remaining mixture was incubated at 27 °C for a further period of 90 min. At the end of 180 min, 0.2 mL of the incubated mixture was treated with 1.0 mL of isopropanol to arrest the reaction. The precipitated proteins in all tubes were separated by centrifugation, and the free cholesterol contents in the supernatant were estimated. The activity of LCAT in the plasma/liver was expressed as a function of the disappearance of free cholesterol during the incubation period.

### 4.5. Oil Red O Staining

The heart tissues stored at −80 °C were taken out and embedded in freezing medium and frozen using a −80 °C freezer. The frozen samples were cut on a cryostat microtome and placed onto microscopic slides. The sections were stained with Oil red O (Abcam, MA, USA) (Catalog number: ab150678) and then observed under a light microscope.

### 4.6. Western Blot Analysis

Protein extracts from the whole heart were obtained following the homogenization of heart samples in radioimmunoprecipitation assay (RIPA) buffer (Merck Millipore, Burlington, MA, USA, catalog number: 20-188), and the homogenates were centrifuged at 4 °C for 30 min at 14,000 rpm. The supernatant was mixed with Laemmli sample buffer (Bio Rad, Hercules, CA, USA, catalog number: #161-0747) and 2-mercaptoethanol (Sigma Aldrich, St. Louis, MO, USA, catalog number: M6250). The samples containing equal amounts of protein were separated by gel electrophoresis. The samples were then transferred onto polyvinylidene fluoride (PVDF) membranes (Thermo Scientific, Rockford, IL, USA, catalog number: 88518) and then incubated overnight at 4 °C with antibodies against HMG-CoA-R (1:1000) (catalog number: sc-271595) (anti-mouse; Santa Cruz., Dallas, TX, USA) and LCAT (1:1000) (catalog number: ab109417) (anti-rabbit; Abcam, Cambridge, MA, USA), whereas β-actin (1:5000) (catalog number: MAB1501R) (anti-mouse and rabbit; Merck Millipore, City, Burlington, MA, USA) was used as a loading control. The samples were then incubated with their corresponding secondary antibodies for 1 h at room temperature, and the proteins were visualized using an enhanced chemiluminescence Pico kit (Thermo Fisher Scientific, Rockford, IL, USA, catalog number: 34580). The signal intensity (densitometry) of the bands was measured using Image J software.

### 4.7. Estimation of Protein in the Heart

The estimation of total protein content in the heart homogenate was performed using a Pierce™ bicinchoninic acid (BCA) assay kit (Thermo Fisher Scientific, Rockford, IL, USA) (catalog number: 23225).

### 4.8. In Vitro Reducing Power of NKT

In an in vitro assay, the reducing power of NKT was estimated using a previously described method of Oyaizu [53]. Varying volumes of NKT (10, 20, 30, 40, and 50 μM) were mixed with 2.5 mL of phosphate buffer (0.2 M, pH 6.6) and 2.5 mL of potassium ferricyanide (1% *w*/*v*). The reaction mixture was incubated at 50 °C for 20 min. Then, 1.5 mL of 10% trichloroacetic acid (TCA) was added and centrifuged at 3000× *g* for 15 min. Finally, 0.5 mL of the supernatant was mixed with 1 mL of distilled water followed by the addition of 0.5 mL of ferric chloride (0.1% *w*/*v*). The absorbance was measured at 700 nm against double-distilled water used as a blank. Increased absorbance of the reaction mixture indicates the increased reducing activity of NKT. The reducing activity of NKT is directly proportional to its antioxidant power.

### 4.9. Statistical Analysis

The obtained results were analyzed using one-way analysis of variance (ANOVA) followed by Duncan’s multiple range test (DMRT). Statistical Package for the Social Science (SPSS) version 17.00 was used in the present study. The results were expressed as the mean ± standard error of the mean (SEM) for statistical comparisons. The statistical significance was considered for *p*-values < 0.05.
